# An Essay on the Philosophy of Medical Science

**Published:** 1846-06-01

**Authors:** Elisha Bartlett

**Affiliations:** Professor of the Theory and Practice of Medicine in the University of Maryland


ART. II.—*An Essay on the Philosophy of Medical Science* By ELISHA BARTLETT, M. D.; *Professor of the Theory and Practice of Medicine in the University of Maryland.*

This work has been before the medical public nearly two years, sufficiently long to have its merits discussed, and to some extent settled by contemporaneous opinion—not always a safe criterion by which to estimate the real, enduring value of a literary or scientific production. The critical notices which we have met with in periodicals of either hemisphere, agree in according to it praise, as evincing philosophical reflection, a candid spirit, and containing wholesome truths—truths which it is of not a little practical importance should be carefully pondered by the medical practitioner. In this general view of the character of the work we desire to express our hearty concurrence. We tender our congratulations, (tardily from necessity) as one of the medical public, on the addition to our slender stock of original literature, of a publication which we believe to be creditable both to the author, and to the profession of this country.

With these acknowledgements, we must express an opinion that several of the fundamental portions maintained by Dr. B. in his Exposition of the Philosophy of Medicine, are unsound and illogical. We propose to adduce some of our reasons for this opinion, designating the doctrines which particularly appear to us erroneous, and endeavoring, frankly, to explain our objections. This is due to ourselves, as well as to the learned author, after venturing to dissent from his views; but more than to either, is it due to the subject, for it will not be denied that to settle upon a firm basis the true philosophy of medical science, is a desideratum of importance, as it is an undertaking difficult to accomplish to the satisfaction of every mind. In commencing this partial review, we are reminded that we must confine our remarks within a limited space. The considerations connected with the points to which we propose to direct attention, are of a character to lead unawares into a wide field of discussion. This must necessarily be avoided, and, in order to economise space, we must condense as much as possible, and sometimes content ourselves with giving a brief statement of the views which we shall criticise, in our own language. It would on some accounts be more agreable and satisfactory always to allow the author to speak freely in his own terms, but we certainly have no disposition other than to represent his views as candidly as possible.

Prof. Bartlett embodies his ideas of the general principles which should preside over scientific investigations in a series of formal propositions. To these, in the main, we find no cause to object. They are, in general, in so far as we may be permitted to judge, in consonance with philosophical rules held in the light of truisms by the logicians of the present day. In the abstract they have the force of self-evident propositions, however much they may be disregarded in their practical applications. Being very often, to say the least, but imperfectly applied, they can hardly be too often, or too forcibly brought before the attention of those engaged in scientific inquiries.

These general truths the author appears to consider applicable to the investigation of vital phenomena in the same mode and degree, as to the study of the physical sciences. To quote his words "there is no essential difference between the philosophy of physical and that of physiological science." That the several principles of logic are the same essentially in every application, will of course be admitted. They relate to the conduct of the understanding in its pursuit after truth, and, hence, to say that a logical method shown to be sound in any instance can be false in any other instance to which it is applicable, involves an absurdity. But that there is no material distinction between the practical study of the phenomena of life and the phenomena of physics, is, assuredly, not true. In other words, vital phenomena present certain strong points of difference in character from physical phenomena; and these points of difference are to be considered in appreciating the application of philosophical methods of investigation to the former. This aspect Prof. B. does not seem to consider, but, on the contrary, he appears to assume an entire correspondence of both classes of phenomena as subjects of study. He says "these doctrines (i. e. the doctrines composing the philosophy of physical science,) with certain modifications in one or two particulars, are identical with the true doctrines of the philosophy of medical science." Of the modifications to which he refers he does not specifically speak, and the inference to be drawn from the remark is, that they are of a trifling, unimportant character, and do not affect the entire applicability of the doctrines of physical philosophy to the investigation of vital phenomena. This is his point of departure in the exposition of medical philosophy, and the subsequent positions in a great measure lean upon it for support. Now it is an obvious truth that the phenomena, or events, manifested in the living organism, possess several peculiar characters, distinguishing them widely from physical events, which pecularities very materially affect the successful application of the rules of investigation which are, comparatively speaking, so easily applied to the physical sciences. We will not undertake here to unfold these distinctions. This would involve an extensive, although as we regard it, a very important department of medical philosophy. We may refer, however, to one point which perhaps affords the most striking and radical distinction: which is the want of precise uniformity in similar phenomena. This is a seeming paradox, but it is nevertheless true. A vital fact occurring at one time, is not an exact representative of the same generic fact at other times. They present variations which cannot be collectively determined, nor individually estimated. In other words, the phenomena of life involve direct or contingent elements which cannot always be appreciated, and which defy all rigid calculations. How different in this respect the phenomena of inanimate nature. The language of Bichat on this subject has lost none of its appropriateness and force. We beg leave to quote: "Physical laws are constant and invariable, they are subject neither to augmentation nor diminution. A stone does not gravitate to the earth with more force at one time than another; in every case marble has the same elasticity, &c. On the other hand, at every instant sensibility and contractibility (vital properties) are increased, diminished or altered: they are scarcely ever the same. We calculate the fall of a heavy body, the motions of the planets, the course of a river, the ascension of a projectile, &c.: the rule being once found it is only necessary to make the application to each particular case. Thus heavy bodies fall always in a series of odd numbers; attraction is in the inverse ratio to the square of the distance, &c. On the other hand, all the vital operations are susceptible of numerous variations. They are frequently out of their natural state. They defy every kind of calculation, for it would be necessary to have as many rules as there are different cases. It is impossible to foresee, predict or calculate anything with regard to their phenomena—we have only approximations to them, and even these are often very uncertain."

Other points of view might be instituted, in which peculiar attributes of the phenomena of life would be equally manifest, but we forego further prosecution of this topic. The aspect under which we have regarded it in the few remarks which have been offered, will suffice to show that Physiology and Pathology, as philosophical pursuits, are not in all respects in the same category with the departments of physical science. We would not be understood to affirm that they require a different system of logic; but what we mean to say, is, that in their relations to logical methods of investigation, certain considerations peculiar to themselves are involved, which cannot be overlooked or disregarded in treating of the philosophy of medical science. And the fact that these considerations are inadequately considered, and, indeed, almost shut out of view in the essay of Prof. B., we cannot but regard a serious blemish, although, as will be seen presently, it was in a measure necessary to the development of certain views which he subsequently presents, and which we shall endeavor to show, are erroneous.

Prof. B. submits a new definition of the Baconian or inductive philosophy, which, as he thinks, expresses more correctly the true scope and methods of this philosophy. He takes occasion to repeat this definition very frequently in the course of his essay, and attaches to it evidently not an inconsiderable degree of importance. It is this: "*All Science consists exclusively in Phenomena and their relationships classified and arranged.*" In the phraseology of this definition we have an inkling of the particular application which is to be made of it to the "principles of medical science." He argues that a confused or imperfect apprehension of the Baconian philosophy is common, and he is disposed to attribute this in some measure to wrong ideas connected with the expression, *inductive reasoning*—he thinks it would be better to allow this phrase to disappear. Let us examine for a moment, this definition, and the erroneous impressions to which he refers; and, perhaps, it will appear that the common ideas on the subject are, after all, nearer the truth than those which he would prefer should be substituted in their place. We will here give the authors words respecting the confused apprehensions just referred to. He says, "The confusion to which I allude is this. There seems to be a common feeling that the facts, phenomena and events, with their relationships classified, and arranged, constitute, not the entire science to which they belong, but only the *foundation* of the science. There is a feeling that these facts and relations are to be used as elements out of which the science is to be built up, or constructed, by what is called *inductive reasoning*. The feeling implies, and the avowed doctrine growing out of it often asserts that the science is *in this subsequent process of reasoning*, and not in the facts themselves, and their relationships. * * * Now what I wish to insist upon is this; that the science *is in the facts and their relationships classified, and arranged, and in nothing else.* The ascertained facts and their relationships, classified and arranged, constitute in themselves, and alone, the science, and the whole science to which they belong. * * * The only reasoning that has any thing to do with the matter, consists simply in the act *of arranging and classifying the phenomena* and their relationships, &c." The author takes frequent occasion to reiterate essentially the same remarks.

We do not dispute the correctness of the formula in which he chooses to embody the ends and means of the Baconian philosophy, although, as a matter of taste, we do not think it is an improvement; but the impression to be conveyed by the tenor of his remarks is, that processes of reasoning have very little to do in this philosophy. The reader is left to infer that science is little more than a mere aggregation of facts; that when facts are brought into juxtaposition they unite from instinctive relationships, or inherent spontaneous affinities, without the intervention of the human mind as the connecting medium. Indeed that it is a serious error which prevails, to suppose that reasoning is the instrument by which scientific truths are unfolded. But is it always an easy task to classify and arrange phenomena? The phenomena now classified under the generic term gravitation, waited for the mind of a Newton to be so arranged. The facts had been always before the senses, and there had been many philosophical observers before Newton's day; but—what was wanting? plainly the reasoning, or, to use the significant expression, the inductive process. No new phenomena were created, but a vast number of far distant phenomena were brought together—inducted—and the intellect of Newton detected the connecting link. The *relationships* of phenomena which Prof. B. every where mentions as if independent of ratiotination, what is it which detects and demonstrates these? What but observation, experience, and experiment, guided always by reasoning! There cannot be any true ground for an issue between processes of reasoning and philosophical investigations. No doubt science has much to suffer, as it has already suffered much, from misapprehension and the misapplication of logical methods; but false reasoning is one thing, and no reasoning another. The advantages of the Baconian reformation in philosophy consisted in the proper regulation of the reasoning powers, and their proper direction for the investigation of truth: and just in proportion to the amount of ratiotination, properly regulated and directed, has science advanced, and will it continue to advance. It is owing to imperfections of human reasoning that science is imperfect. No one will assert that the "*relationships*" of natural phenomena will ever be completely unfolded, or that their "*classification and arrangement*" will ever be perfect—and why will they not? Not because the phenomena themselves do not admit of such systematic investigation, but because our reasoning faculties are limited and liable to error. Adopting, then, the definition which the author prefers, of physical philosophy, to our mind it involves precisely those operations of the reasoning faculties which are commonly, and in our estimation better expressed by the terms *induction* and *generalization.* As we have before intimated, the idea suggests itself that the phraseology of the definition is adapted especially to suit the method of investigating medical facts which it is the author's object subsequently to establish, viz:, the *numerical method.*

We are, also, disposed to take issue with the author in his next position, viz., that *physical science is the result of observation.* We think this statement is too broad. Here, too, he is of opinion that "*ideas*, and *reasoning*, and *deduction*, are supposed to have much more to do with it [observation] than is really the case." We do by no means deny that physical science is the result of observation, but it is the result of observation and *something more*, viz. the processes of reasoning. Mankind in all ages had observed that heavy bodies fell to the earth, and had watched the motions of the heavenly bodies, but this never led to the discovery of the law of gravitation, until Newton suspected and demonstrated the relationship. We shall not, of course, be understood to disparage the value of observation; we acknowledge its importance as fully as Prof. B., but we do not consider it adequate in itself to constitute science. We will not, however, enlarge upon this point. Either we are so thoroughly imbued with the popular prejudice in favor of ratiotination of which he speaks, that we cannot take a right view of the matter, or the view which we have thus briefly presented, is so obvious as not to need farther illustration.

Our strictures thus far have related to the first part of the essay, which is entitled the "Philosophy of Physical Science." A chapter devoted to the "nature of hypothesis," affords room for criticism. The position that "all science is absolutely independent of hypothesis," in our view is altogether too unqualified. We believe that if we could analyse the intellectual processes which have led to great discoveries, we should find that nearly all pre-existed in the shape of hypotheses before becoming established truths. Reason generally forsees the results of demonstration; and in prosecuting scientific investigations it is not necessary that we discard all hypothetical expectations, but only that we require, both of ourselves and others, rigid proof before we assume or receive a hypothesis for established fact.

We pass now to the second part, viz, "THE PHILOSOPHY OF MEDICAL SCIENCE." We are warned by the length to which our remarks have already extended of the necessity of brevity in those which remain. We shall endeavour to condense what we have to say in as few words as possible. Of a considerable portion of the second part, we can only speak in general terms of commendation, recommending those who may honor this brief notice with a perusal to consult the work itself, assuring them that they will find both pleasure and profit in so doing.

Having considered the several divisions of medical science into Anatomy, Physiology, Etiology, Pathology, Diagnosis and Therapeutics, Prof. B. devotes a short chapter to these propositions: "*Our knowledge of anatomy not dependent upon our knowledge of other branches of Medical Science. Our knowledge of one branch of anatomy does not include the knowledge of any other branch.*" To these propositions we enter no disclaimer. As a branch of natural history, strictly, being concerned exclusively with sensible properties, Anatomy is a study of pure observation, and, of course, is limited to the appearances presented by the body collectively, and separated into its various component parts.

We pass to the next chapter in which the following position is advocated: "*Our knowledge of Physiology is not deducible from our knowledge of Anatomy.*" True it is not deducible; that is to say the Science of Physiology could never have been brought to its present condition by examining the dead body alone, nor, it may be added, could it ever have been what it is without an examination of the dead body. Here, however the author admits as a qualification the doctrine of *final causes*, He considers this a legitimate branch of physiology in the relation which physiology sustains to anatomy, and this, in fact, covers the whole ground. Conceding this, we presume Prof. Bartlett's views with reference to the connection of the two departments are essentialy those which are generally entertained upon the subject.

The next proposition claims some discussion. It is that "*our knowledge of Pathology is not deducible from our knowledge of Physiology.*" This he takes occasion to repeat, and state as broadly as possible, to wit: [the italics being his own] "*Pathology is not founded on physiology. The latter is not the basis of the former. The one does not flow from the other. Our knowledge of the one does not presuppose our knowledge of the other.*" He subsequently admits some slight qualifications, which, to use his own language, are "more nominal than real," and they "reduce themselves when closely examined and analyzed, within very insignificant dimensions." We cannot discuss the points involved in this position as their importance merits. It would require more space than we can appropriate to a brief review. We must content ourselves with advancing some thoughts which may perhaps serve as suggestives to a proper train of reasoning in the minds of our readers.

What is Physiology? It is the science of vital phenomena; in other words the science which investigates the actions and operations of the living organism. Now what is Pathology? Is *it* not the science of vital phenomena? Is *it* not engaged with the investigations of actions and operations of the living organism? The two branches of study are, then, in fact, occupied with the same class of phenomena or events. But there is a distinction — what is it? The distinction consists in the fact that the phenomena are studied under different conditions of manifestation. The one studies them in a state of health; the other in a state of deviation from health. Now if one undertake to determine accurately the dividing line separating health and disease he will not find it an easy task. Practically, and as a matter of personal consciousness, it is generally not difficult, but it is not easy to make a rigid definition. This is further confirmation of the fact that they are but separate departments of one science. The division is, in fact, to a certain extent arbitrary, and chiefly useful because of its practical convenience.

These considerations seem to us to place the matter in its true light, and enable us to consider each pursuit in its true relation to the other. We cannot strictly deduce one class of facts in Physiology from another class. Digestion is one thing, the excretion of urine another; we cannot deduce the facts appertaining to the urinary secretion from the facts appertaining to digestion, although science enables to institute certain important relations between these two functions. So with regard to physiological and pathological facts generally, when they are compared together; we have no right to infer the latter from the former, irrespective of observation of the latter separately; and yet, they are often closely associated, and each borrow reflected light from the other. Each constitute a division of one comprehensive Science, and they both mutually assist and sustain each other.

This general view, which we rely upon the reader's reflection and good sense to carry out, and substantiate by illustrations which will readily suggest themselves, is incompatible with the assertion of Prof. B. that there is in effect little or no connection between them. In our estimation he fails entirely, in his treatment of this subject, to substantiate his positions. He adduces the instance of inflamation, and asks if we should have been able to deduce the phenomena and facts belonging to this morbid process from our physiological knowledge alone. We reply, at once, No: but, then, we would enquire in our turn, should we have known as much as we do of this process, if we were totally without the light of physiology; and is it not highly probable, that if we knew more of the vital actions and properties inherent in the healthy condition of the tissues, we should be better able to explain, than we are, the phenomena which characterise the inflammatory state?

There is another view of this subject; viz., that afforded by the actual history of the progress of medicine. Have the developments of physiological truth been of no value to pathology? Would the latter have been what it now is, all other things being equal, without the discovery of the circulation by Harvey? Has pathology gained nothing from the modern elucidation of the separate functions exercised by different portions of the nervous system? Or, to take one more illustration, recent investigations have shed considerable light on the physiologo-chemical relations existing between the secretion of urine and the processes of assimilation and dis-assimilation. What has been the effect upon pathology? Does not the urinary excretion afford at this time a very important series of facts involved in pathology and diagnosis, which, until lately, were entirely overlooked or disregarded? With these enquiries we leave this branch of the subject, and proceed to the next chapter.

The subject of this chapter is Etiology. Here the author advances the position that "*our knowledge of the causes of disease results exclusively from observation.*" We pass by this without comment, although the position, in our view, demands qualifications not less than those already noticed.

The next chapter relates to Therapeutics. Here he divides writers and practitioners into two classes, the *rationalists* and the *empirics*. The terms, with the significancy usually attached to them, sufficiently express the grounds of distinction. The author enters his unqualified protest against the former, and expresses his exclusive reliance upon empiricism as the only true doctrine of therapeutics. The following quotation is given by the author in italics for greater emphasis: "*Therapeutics is not founded on pathology. The former cannot be deduced from the latter. It rests wholly upon experience. It is, absolutely and exclusively an empirical art.*" Prof. B. is certainly entitled to the merit of taking a bold and decided stand. This we are glad to see. It makes the issue plain and clear.

We entertain no doubt however, that Prof. B. disproves practically his own statements each day that he performs the duties of a medical practitioner. Does he whenever he prescribes a cathartic, laxative, emetic, anodyne, &c., in the routine duties of his profession, rely exclusively upon evidence that in precisely the same catenation of circumstances these remedies have been found by experience to relieve the patient; or does he not, also, and more especially consider that the inductions rationally, as well as empirically, call for the particular remedies which he selects? If he will carefully and impartially analyse the operation of his own mind in determining upon remedies in particular cases, we are willing to rest with this *argumentum ad hominem.*

It is true that we have a class of remedies which are exclusively empirical. They are so, because science has not yet succeeded in unfolding the rationale of their operation. Such are arsenic, quinia, mercury, iodine, &c., which the author adduces. But who can doubt that if the *modus operandi* were rationally explained, we should be far better able to employ these remedies with safety and advantage, than we now are? As it is, in directing their administration, do we not always act upon rational indications as to the time of administration, the doses, &c.? Most assuredly this is the case.

The truth is, *empiricism* and *rationalism* should not be set against each other in therapeutical investigations. They are both susceptible of logical and useful application; and science requires their conjoint operation. The latter will serve to direct and regulate the former. We admit that it does not suffice of itself, but requires the evidence of the former to confirm its conclusions. The former, when it is entirely applicable, is more reliable than the latter: It may be more safely trusted singly. Yet, there are few or no cases in which it does not involve the other; and it will certainly be rendered more complete and satisfactory by the union.

The true policy for the medical philosopher is not to rank himself as an exclusive adherent to either side. He should neither be a *rationalist* nor an *empiric;* but he should be both. Eclecticism here is not the safest and best rule; but he will succeed best who, within just and philosophical limits, recognizes both as avenues to the elucidation of truth, and the acquisition of knowledge.

We pass by the chapter on Diagnosis, not because it does not contain matter for comment, but because we must hasten to the conclusion of our remarks.

We come now to the kernel of the philosophy—the positions which have been previously advanced, are intended merely as the foundation, and the pedestal; the column thereupon to be erected is in honor of the *numerical system.* The reader is supposed to be conversant with the methods and the scope of this system. Its relations to the several points which the author endeavors to establish, have doubtles been already apparent. These points are fundamental, and essential to the support of the system. Without them, the system, as an exclusive system, or, in other words, as constituting the philosophy of medical science, necessarily falls.

Were we to enter upon a discussion of the merits of this system of medical investigation, we should certainly require the entire space which we can appropriate to this article. But we do not believe that its discussion is at this time called for. It has been before the profession for several years, and its capabilities have occupied not a little share of periodical medical literature. It is several years since we ventured to communicate some reflections on the proper place which it should occupy as a department of medical philosophy. [New-York Quarterly Journal of Med. and Surg. for April, 1841.] Farther reflection and observation have not led us to believe that it is ever destined to take the place of other methods, or that it will ever render medicine an exact science. In the first place, its application is confined within narrow limits. It will only apply to those diseases which are susceptible of ready and clear diagnosis, such as pneumonitis, pleuritis, &c., and which present similarity in a large number of phenomena. And then, to arrive at numerical results sufficient for all the purposes of science, the comparisons must be infinitely varied. We concede that very great advantages have been, and remain to be derived from statistical comparisons. Science is greatly indebted to Louis for the introduction and successful application of this method. We should all, as medical observers and practitioners, adopt it; but it can never supersede rational investigations carried on independently of arithmetical computations.

In the next place, supposing that we possessed statistical facts of pathology and therapeutics to as full an extent as is conceivable—to a far greater extent than is practicable—the practice of medicine involves not only the knowledge of general rules, but the application of these rules to individual cases. We have, in every case, to determine in how far the general rules will apply, and in how far they are modified by the particular circumstances of the case. Useful as it is to know what are the probabilities of the case, they will not suffice. Medical practice involves quite as much the *exceptions* which are disregarded in the law of probabilities, as it does the laws of probabilities—aye, much more. Nothing can remove the necessity of studying each case of disease as a problem in itself, or rather a series of problems, varying with its progress every day and hour. The analogy of the principle of life assurance, furnishes the readiest mode of illustrating the fallacy of this system as a system susceptible of complete and individual application. The life assurance office bases its policies on the probabilities of human life which have been very accurately determined. It is a clear problem how much it is worth *per centum* to insure a multitude of lives, the ages being given. So it may be practicable to ascertain, by enumeration, what will be the ratio of mortality from a certain disease under different methods of treatment. But in the case of life assurance, who goes to the assurance office to inquire how long he is to live? And in the instance of the disease, how can we infer from the general numerical results the efficacy of treatment in particular cases?

With these discursory remarks, we leave the subject; and, at the same time, take leave of the author. In so doing, however, in justice to the merits of the work, and our own appreciation of them, we must remind the reader that we have selected those portions for comment which appeared to us open to criticism, leaving unnoticed much the larger share of the work. The medical student, and the practitioner, will find among the latter much valuable truth — truth which it is of not a little importance should be more considered, and practically regarded, than is apt to be the case. We commend to the careful attention of the reader the account of the various prominent medical doctrines which have had their day, or continue to be more or less in vogue, and the observations therewith connected. The considerations pertaining to the employment of the numerical system, are, also, highly important. They may be appreciated without coinciding with the author in regard to this system as the only method consonant with principles of midical philosophy. Indeed, most of the propositions which we have criticised contain much truth — and important truth. The objections chiefly apply to their exclusive and unqualified character.

We trust that the author will continue his valuable contributions to American medical literature.

